# Suicides in Visually Impaired Persons: A Nation-Wide Register-Linked Study from Finland Based on Thirty Years of Data

**DOI:** 10.1371/journal.pone.0141583

**Published:** 2015-10-28

**Authors:** Victor Benno Meyer-Rochow, Helinä Hakko, Matti Ojamo, Hannu Uusitalo, Markku Timonen

**Affiliations:** 1 Department of Biology (Physiology), Oulu University, (V.B.M-R), Oulu, Finland; 2 Department of Psychiatry, Oulu University Hospital, Oulu, Finland; 3 Finnish Federation and Register of the Visually Impaired, Helsinki, Finland; 4 Department of Ophthalmology, University of Tampere, Tampere, Finland; 5 Center for Life Course Epidemiology and Systems Medicine, University of Oulu, Oulu, Finland; University of Vienna, AUSTRIA

## Abstract

Focusing on seasonality, gender, age, and suicide methods a Finnish nation-wide cohort-based study was carried out to compare suicide data between sighted, visually-impaired (WHO impairment level I-II, i.e., visual acuity >0.05, but <0.3) and blind (WHO impairment level III-V, i.e., visual acuity <0.05) victims. Standardized mortality ratios (SMR) of age- and gender-matched populations from official 1982–2011 national registers were used. Group differences in categorical variables were assessed with Pearson's Chi-square or Fisher's Exact test and in continuous variables with Mann-Whitney U-test. Seasonality was assessed by Chi-square for multinomials; ratio of observed to expected number of suicides was calculated with 95% confidence level. Hanging, poisoning, drowning, but rarely shooting or jumping from high places, were preferred suicide methods of the blind. Mortality was significantly increased in the visually impaired (SMR = 1.3; 95% CI 1.07–1.61), but in gender-stratified analyses the increase only affected males (1.34; 95% CI = 1.06–1.70) and not females (1.24; 95% CI 0.82–1.88). Age-stratified analyses identified blind males of working age rather than older men (as in the general population) as a high risk group that requires particular attention. The statistically significant spring suicide peak in blind subjects mirrors that of sighted victims and its possible cause in the blind is discussed.

## Introduction

Visual impairment and blindness as risk factors in suicidality have received mixed acceptance in the past: associations were apparent in combination with depression and non-ocular conditions like poor general health and physical illness [1–3]. However, direct effects of blindness in a study of 200 suicide deaths only resulted in an elevated, but not significant hazard ratio (HR = 1.50; 95% confidence interval 0.90–2.49) and it was concluded that visual impairment “…may be associated with an increased risk of suicide through its effect on poor health” [4], or in connection with those suffering gradually worsening sight, associated with the expected disability [5]. In an otherwise comprehensive list of suicide risk factors visual impairment was not mentioned [6] and Scuderi et al [7] reported recently that there was “no strong evidence that measures of visual impairment were linked to emotional well-being and depression”, although depressive disorders are commonly considered severe risk factors [6,8].

Every 40 seconds someone somewhere in the world commits a suicide [9] and Finland, a Nordic country of 5.5 million inhabitants and a population density of 14/km^2^, has one of the highest suicide rates in the world. According to the 2012 Finnish Statistical Yearbook, 24.6 males and 7.9 females per 100,000 inhabitants committed suicide [10]. Unsurprisingly, risks of developing suicidal ideation and self-destructive behaviour for different sections of the population were assessed and analysed in a series of enquiries, e.g., focusing on persons 25 years of age or younger [11,12], persons older than 65 years of age [13], persons suffering from rheumatoid arthritis [14] or atopic disorders [15], and persons with physical or mental illnesses [16–19]. In agreement with results from other countries [8,9], it has become abundantly clear that irrespective of the sub-section of the population under scrutiny, suicide attempts and depressive disorders frequently precede a suicide, significantly more men than women kill themselves and suicides peak during the spring and early summer months in countries with temperate climes [20–24].

Yet, a cohort-based study of suicides, focusing exclusively on the visually-impaired and blind has neither been undertaken in Finland, nor anywhere else in the world. This dearth of information is regrettable as 285 million people are estimated to be visually impaired worldwide: 39 million are blind and 246 have low vision [2]. In Finland out of a population of 5.4 million in 2012 roughly 80,000 (according to the Finnish National Institute of Health & Welfare, with approx. 50,000 visually impaired people in the age group 65+ alone) were considered visually-handicapped [25]. With suicide data available for seeing and visually-impaired victims for the years 1982 to 2011, covering the entire country, we found ourselves in an excellent position to carry out a population-based comparison of suicides between sighted and visually impaired victims. We focused on seasonality, gender, age, and suicide methods, because earlier studies of Finnish suicides in which visually impaired victims were not separated had suggested that (a) the spring/early summer suicide peak was related to photoreception [15,23,26,27], and (b) in agreement with the worldwide situation considerably more men than women killed themselves [8,9–11] while suicide risks increased with age being “highest in persons aged 70 years or over” [28]. We also felt it would be important to know which methods the visually impaired used to commit suicide, allowing perhaps preventive measures to be more successfully employed.

## Materials and Methods

### Study design

The Finnish Register of Visual Impairment is maintained by the Finnish Federation of the Visually Impaired, and is subject to the national Institute for Health and Welfare (THL), which is a governmental office. The operation of the Register is regulated by law. Health care authorities are responsible to forward to the Register basic ophthalmological information on persons with permanent visual impairment based on WHO´s definition of visual impairment [29]. In this study, subjects with a WHO impairment level of I-II, i.e., visual acuity ≥0.05, but <0.3 were defined as having “low vision”, and subjects falling under category III-V, i.e., visual acuity < 0.05, were considered “blind”.

Central statistics illustrating certain socio-demographic variables (e.g., level of education, family status, employment status) of Finnish residents and the degree of visual impairment can be found in the Statistical Annual Report [30]. The Register promotes research on visual impairment by providing specific statistics and by encouraging joint research projects. For the purpose of this study, permission to use data on the subjects with visual impairment was obtained from The Finnish Register of Visual Impairment covering the years 1982–2011, but a linkage to permit the inclusion of more detailed socio-demographic data was not obtained.

Causes of deaths were obtained from the National Cause-of-Death Register provided by Statistics Finland. Finnish Register of Visual Impairment data are linked with this register by unique personal identification codes given to each Finnish citizen. The study was licensed by the National Institute for Health and Welfare (THL) (#39/5.05.00/2012). The Ethics Committee of the University of Oulu approved the study and the Finnish Register of the Visually Impaired as part of the National Institute for Health and Welfare (THL) granted the release of records towards this research work. The data were not anonymised, because linking to other registers had to be possible.

Registration of the visually impaired is compulsory in Finland and regulated by law. Health care authorities are responsible to forward to the Register information on the visually impaired band no permission of the patient is required.

### Suicide cases

Our study sample involved cases classified as suicides (ICD-8 and ICD-9: E950-E958, ICD-10: X60-X84, Y87.0). Finnish law requires that in every case of violent, unnatural, sudden or unexpected death the possibility of suicide must be assessed by the police and by means of medico-legal examinations. The decision to classify a death as suicide is made by a forensic examiner, who compiles the death certificate. All death certificates are recorded in the national cause-of-death register. Forensic definition practices with respect to suicide remained the same throughout the period concerned and were consistent over the whole of Finland. Only completed suicides were considered; information on co-morbidity and data on earlier and unsuccessful suicide attempts were not available.

### Suicide methods

The death certificates include information on the method of suicide. Suicide methods are categorized as violent or non-violent. Violent methods include hanging, drowning, shooting, jumping from a height, intentional traffic accidents and wrist-cutting, while poisoning, gassing and other methods not covered under the violent label are placed in the non-violent category [31].

### Statistical analyses

Statistical significance of group differences in categorical variables was assessed with Pearson’s χ2- test or Fisher’s Exact test and in continuous variables with Mann-Whitney U-test, when appropriate.

Suicide mortality of visually impaired persons was compared with that of the age- and gender matched general Finnish population by using Standardized Mortality Ratio (SMR). Age-, sex-, and cause-of-death specific numbers of the general Finnish population were obtained from the statistical database (StaFin; Health/Causes of Death) of “Statistics Finland” [10]. SMR was calculated as the observed number of deaths divided by the expected number of deaths. The expected number of deaths was calculated by multiplying the number of subjects by the relative proportion obtained by dividing the number of deaths in the whole population (within an age and gender group) by the total number of the population in the corresponding age and gender group. The SMR is statistically significantly elevated when the lower confidence interval (CI) is greater than 1.00, and significantly reduced when the upper CI is less than 1.00.

Cox proportional hazards model was used to examine the difference in time between the onset of visual impairment and suicide between blind persons and persons with low vision. The analysis is adjusted for a person’s age at death. The data of Cox proportional hazards model covers 67 out of 91 suicide cases, since the information on the onset year of visual impairment was not available for 24 suicide cases. In addition, to demonstrate high and low risk periods for suicide after the onset of visual impairment, the time was categorized as: ≤25th (lower) percentile, 25th-75th and ≥75th (upper) percentile.

Overall seasonality was assessed with the χ^2^-test for multinomials and the ratio of observed to expected number of suicides with 95% confidence intervals was calculated [32]. When calculating ratios, adjustment for equal month length was performed and the effect of leap years was taken into account. All statistical analyses were carried out using IBM SPSS Statistics, version 22, for Windows.

## Results

### Subjects’ visual impairment characteristics

Of the 91 cases of suicides committed by the visually impaired, 63 (69%) were committed by people with a WHO impairment level of I-II (“low vision”, i.e., visual acuity ≥0.05, but <0.3) and 28 (31%) by people categorized under the III-V category (“blind”, i.e., visual acuity <0.05). In both groups males dominated (Table 1). Visual impairment was diagnosed at a younger age in blind persons compared with persons in the low vision category. In blind persons, suicide deaths peaked amongst the 40–64 year olds, but in the persons with low vision, most of the suicide deaths involved 65+ year olds.

**Table 1:** Characteristics of suicide victims by category of visual impairment (N = 91).

		Level of visual impairment^a^			
Characteristics of suicide victims		Visually impaired total (WHO I-V: combined)(N = 91)	Low vision (WHO I-II) (N = 63)	Blind (WHO III-V) (N = 28)	Statistical significance of group difference
		N (%)	N (%)	N (%)	P-value^c^
Gender					0.348
	Male	69 (75.8)	46 (73.0)	23 (82.1%)	
	Female	22 (24.2)	17 (27.0)	5 (17.9%)	
Visual impairment age^b^					0.080
	< 1	5 (7.5)	3 (6.5)	2 (9.5%)	
	1–17	4 (6.0)	2 (4.3)	2 (9.5%)	
	18–39	16 (23.9)	10 (21.7)	6 (28.6%)	
	40–64	11 (16.4)	5 (10.9)	6 (28.6%)	
	65+	31 (46.3)	26 (56.5)	5 (23.8%)	
Age at death by suicide					0.064
	20–39	14 (15.4)	8 (12.7)	6 (21.4)	
	40–64	31 (34.1)	18 (28.6)	13 (46.4)	
	65+	46 (50.5)	37 (58.7)	9 (32.1)	

^a^Based on the WHO categorization of visual impairment [29]: low vision (WHO I-II) = visual acuity ≥ 0.05, but <0.3; blindness (WHO III-V) = visual acuity <0.05. In one case the degree of impairment was not known; that person was included in the low vision category.

^b^Data available for 67 subjects

^c^Pearson’s Chi-square or Fisher’s Exact test, two-tailed significance.

### Standardized mortality ratios (SMR)

When compared with age and sex matched individuals of the general population of Finland, the mortality of the visually impaired (N = 91) was significantly increased (SMR = 1.3; 95% CI 1.07–1.61) (Table 2). However, in gender stratified analyses, the SMR was shown to be significantly increased only in males (1.34; 95% CI 1.06–1.70). In age stratified analyses significant SMRs were found in age groups of 30–39 years (2.17; 95% CI 1.13–4.17) and 40–49 years (2.66; 95% CI 1.65–4.28).

**Table 2:** Standardized Mortality Ratios (SMR) for visually impaired persons.

		Number of suicides			
		Observed	Expected^a^	SMR	95 % CI for SMR
Suicides	Total data	91	69.3	1.31	1.07–1.61
Gender					
	Males	69	51.5	1.34	1.06–1.70
	Females	22	17.7	1.24	0.82–1.88
Age at death by suicide					
	20–29	4	3.3	1.22	0.46–3.25
	30–39	9	4.2	2.17	1.13–4.17
	40–49	17	6.4	2.66	1.65–4.28
	50–59	11	7.3	1.51	0.83–2.72
	60–69	8	7.3	1.10	0.55–2.21
	70–79	11	14.5	0.76	0.42–1.37
	80–89	22	21.4	1.03	0.68–1.56
	90 +	9	5.0	1.81	0.94–3.48

^a^Expected number of suicides is calculated based on the age- and sex-matched general Finnish population.

### Seasonality of suicides

**Fig 1:**
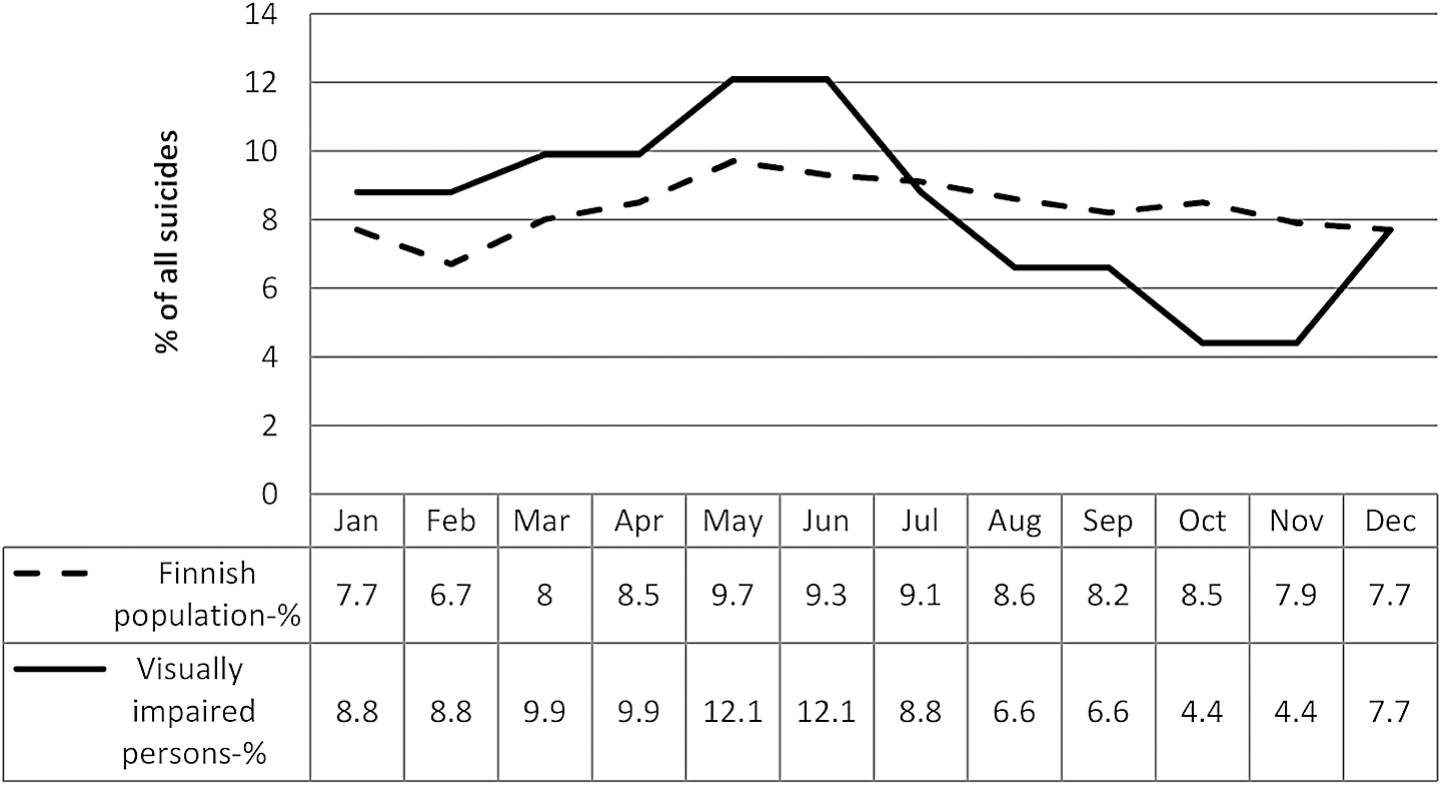
Monthly distribution of suicides of the visually impaired and the general Finnish population.

As seen in Fig 1 the monthly distribution of suicides in visually impaired persons mirrored the monthly distribution of suicides in the general population (χ2 = 6.63, df = 11, p = 0.828) with a characteristic increase in the months of spring and a decrease in winter months.

When seasons (winter, spring, summer, autumn) were examined, no statistically significant difference was found in comparison with the general population either in persons with low vision (χ2 = 4.75, df = 3, p = 0.191) or in the blind (χ2 = 3.16, df = 3, p = 0.368). There was, however, a significant difference in the seasonal pattern of suicides between persons with low vision and the blind (χ2 = 13.44, df = 3, p = 0.004): the spring suicide peak was more noticeable in the blind (39.3%) than in persons with low vision (28.6%).

### Methods of suicide

As shown in Table 3, based on data of all the visually impaired persons (N = 91), the two most common suicide methods were hanging and poisoning; drowning featured as the third most common method amongst blind people, but was rare in the low vision group, in which shooting was more common. Of note is that, jumping from a high place had no representative in the blind group, but it was the fourth most common suicide method in the low vision group of the visually impaired.

**Table 3:** Suicide methods of the visually impaired.

		Level of visual impairment^a^	
Suicide methods^b^	Combined (N = 91)	Low vision (N = 63)	Blind (N = 28)
	N (%)	N (%)	N (%)
Drowning	5 (5.5%)	1 (1.6%)	4 (14.3%)
Gas	2 (2.2%)	1 (1.6%)	1 (3.6%)
Hanging	38 (41.8%)	28 (44.4%)	10 (35.7%)
Jumping from a high place	5 (5.5%)	5 (7.9%)	0
Poisoning	26 (28.6%)	17 (27.0%)	9 (32.1%)
Shooting	11 (12.1%)	8 (12.7%)	3 (10.7%)
Sharp object	2 (2.2%)	1 (1.6%)	1 (3.6%)
Traffic	1 (1.1%)	1 (1.6%)	0
Other	1 (1.1%)	1 (1.6%)	0

^a^ Based on the WHO categorization of visual impairment [29]: low vision (WHO I-II) = visual acuity ≥ 0.05, but <0.3; blindness (WHO III-V) = visual acuity <0.05. In one case the degree of impairment was not known; that person was included in the low vision category.

^b^ Statistical significance of difference in the choice of suicide method between persons with low vision and blind persons, χ2 (8, N = 91) = 10.09, p = 0.211.

### Time between onset of visual impairment and suicide

**Fig 2:**
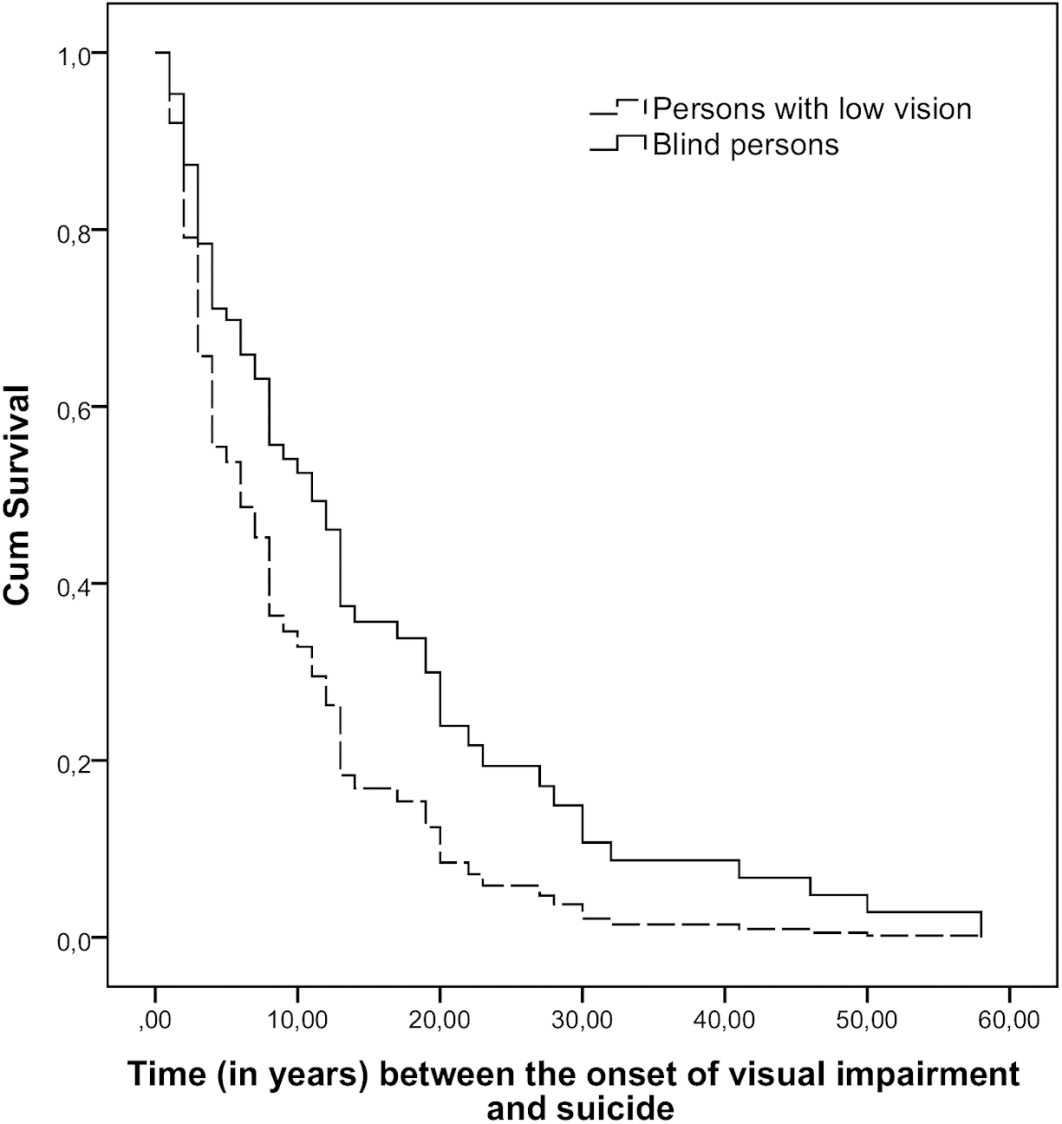
Cumulative survival in relation to onset of visual impairment and suicide.

The results of the Cox proportion hazard model showed a statistically significant difference in time between the onset of visual impairment and suicide between blind persons and those with low vision (HR = 1.73; 95% CI 1.00–2.98) after adjusting for the age at death (Fig 2).

The time (in years) between the onset of the visual impairment and suicide was significantly longer among persons with low vision (median 6, IQR 3–13) than in the blind (median 13, 4–27) (Mann-Whitney U-test, z = -2.31, p = 0.021). Further, to demonstrate risk periods for suicide after the onset of visual impairment, time was analyzed in three categories (≤25^th^ percentile, 25^th^-75^th^ and ≥75^th^ percentile). The proportions of persons belonging to the to the low vision group and the blind, according to the different time intervals between the onset of visual impairment and suicide, varied as follows: 1–3 years, 39.1% vs. 14.3% (χ2 = 4.14, df = 1, p = 0.042); 4–14 years, 41.3% vs. 47.6% (χ2 = 0.23, df = 1, p = 0.628), and 15–58 years, 19.6% vs 38.1% (χ2 = 2.61, df = 1, p = 0.106).

## Discussion

The total number of suicides committed by the blind and visually impaired in Finland, based on our official data, does not seem to be particularly high, representing roughly 0.3% of all suicides per year. We tried to obtain additional suicide figures from more populous countries, but for confidentiality reasons and the fact that few countries separate suicides committed by sighted and visually impaired victims we were unable to include data other than those from Finland. Even so, assuming that the percentages of suicides committed by the blind and visually impaired do not vary greatly in other countries from that of the relatively sparsely populated country of Finland, we can expect to find, for instance, in England and Wales with a population almost exactly ten times that of Finland and a most recent annual suicide figure of 5,140 (versus Finland’s 873), a not exactly negligible number of suicides committed by the blind and visually impaired. Given the 804,000 cases of global suicides in 2012 [28], we should have approximately 2,500 blind and visually impaired victims amongst them when based on the Finnish ratio of blind and visually impaired to sighted victims. If we then expanded the calculation to cover 30 years as with our Finnish data set, we would arrive at a figure of suicides by the blind and visually impaired victims that should certainly be alarming.

Blind people in Finland cannot obtain a license to possess firearms, which virtually rules out shooting as a suicide method. The almost general absence of jumping from high places, traffic accidents, throwing themselves in front of trains etc., but of hanging and poisoning and drowning as preferred methods of suicide in the blind also comes as no surprise and requires no further discussion. Neither does the fact that compared with the totally blind, shooting seems more common than drowning while jumps from high places appear in victims with some, albeit low, visual capacity, but not in the totally blind. We therefore intend to focus the discussion on (a), the significantly increased risk of suicide in blind men of working age; (b), the suicide risk in very old women, and (c), the pronounced spring peak of suicides in the blind.

While suicides in the blind peaked in the 40–64 age bracket, those involving subjects with low vision and those of the general population peaked in victims 65 years of age or older. However, there exist clear differences with regard to gender and age, which could explain why earlier studies on visual impairment and blindness as risk factors in suicidality have been inconsistent [1–5,7]. Our gender-specific analysis of standardized mortality ratios showed no statistically significant risk increases for women of all age groups compared with the general population, but a significantly elevated risk for visually impaired men.

This difference between male and female subjects becomes even more apparent, when we consider life expectancies of men and women in Finland and the age structure of new registrations of persons with visual impairments in the Finnish Registry of Visual Impairment. A Finnish woman’s life expectancy is 6.3 years longer than that of her male compatriot and since the average age of newly registered visually impaired persons is 83 years, 61% of the new registrants are women [30]. Consequently the number of registered female visually impaired people at age 85+ is 3.3 times that of the registered visually impaired males. Yet, blindness across all age groups is somewhat more prevalent in men than in women (i.e., 25% and 18%, respectively) and although women aged 70+ may need to be considered vulnerable with regards to suicide overall [28], we could not convincingly demonstrate that specifically women of ages 80+ with low vision showed an elevated suicide risk.

Considering the Finnish population as a whole, 18% of all suicides involve people 65 years of age or younger, but singling out the visually impaired our study shows that the figure involving them jumps to 50%. Reasons for this much higher percentage are likely feelings of depression [33,34], resulting from loneliness and helplessness due to the physical handicap of the visual impairment as well as possibly other ailments and a feeling of hopelessness, the latter having been identified as a severe factor in victims with multiple earlier suicide attempts [35]. Regrettably, the data available for our study did not contain information of previous suicide attempts. It has, however, also been reported that, for example, patients with open-angle glaucoma, perceiving their own disability, did indeed display affective temperaments associated with higher hopelessness, but the results of that study [7], discordant with other studies [36,37], also indicated “that objective indices of visual impairment may not be associated with poor well-being and depression”. Therefore, other factors need to be considered.

There is, for instance, the economic stress to make ends meet when incomes are inadequate and in low and middle income countries those at risk (even if not visually impaired) also find themselves in the bracket of the 30 to 49 year olds in contrast to figures from the more affluent countries [28]. As in Finland only 40% of the visually impaired in the working age group are employed, but with an annual income of on average 26,400 Euros then earn considerably less than their seeing counterparts (Ojamo, unpublished), some economic hardship and associated stress can be expected [38]. Given the skewed age structure of the adult population with visual impairments, we identified blind men of working age (i.e., men who because of their handicap cannot easily realize their career dreams and face greater difficulties in competing for jobs than their sighted peers) as a particularly high risk group. Very old (80+) women with low vision have to be seen as vulnerable as those without any visual impairment and on account of their advanced age are also in need of heightened attention in society.

Turning now to the suicide peak in spring, it may at first seem surprising to find a pronounced and significant annual peak in the suicides of the blind that is coincident with that of sighted suicide victims, for whom spring peak suicides have been well documented [20–24,39]. Numerous hypotheses have been put forward to link the rise in suicides during spring and early summer in the seeing population to environmental factors, in particular the increasing daylengths and brighter conditions, which affect the endocrine system and involve melatonin as a biorhythm coordinator [22,40]. The question is how blind people could be affected by the increasing spring brightness if they cannot see.

Even totally blind people, however, may be able to perceive the dramatic changes in brightness accompanying the arrival of spring in high latitudes with daytime versus night-time durations increasing by 10–20 minutes (depending on the latitude) from day to day. Measurements by Wan et al. [41] have shown that transmittance of light through the human skull increases progressively with wavelengths from 600–814 nm and that even blue light “less than 500 nm can reach the brain”, albeit reduced to 10^−5^–10^−4^ of the original intensity.

Whether or not such low light levels are capable of triggering the rhythmicity of the release of the pineal gland’s melatonin is unknown, but blind subjects on account of their inability to see have to cope constantly with disturbances in their melatonin secretion, suffering sleep disruptions [42] and circadian disorganization [43]. Longer days, brighter lights or the recently suggested atmospheric pressure changes [44] as well as a multitude of other factors, including social ones, could combine to aggravate sleep disturbances, the latter associated “with risk for death by suicide” [45] and a rise in C-reactive protein levels [46]. An increase of the latter had been shown to increase the probability for severe current and recurrent depressive episodes especially in male subjects [47].

Suicide figures of the general population in Finland have gone down for all age groups in recent years [13], but whether this trend, thought to be the result of greater vigilance in detecting people at risk and an increased use of antidepressants in combination with general improvements in taking care of people at risk, was paralleled by suicides committed by the visually impaired cannot be ascertained on account of the small data base available for the latter. Although the Social Insurance Institution (KELA) offers full rehabilitation services and special technical aids to visually impaired children and youngsters as well as some support to their families (and this, too, could have helped bring down suicides), those beyond working age miss out on these services. To provide suitable occupation with adequate remuneration to blind and visually impaired working age men, would increase their self esteem and very likely prevent them to see suicide as an option. For the blind and very old women heightened supervision and care are imperative, so that access to potentially lethal drugs and poisons will be strictly monitored.

Currently Finland’s central hospitals (a total of 22 in the country) provide guidance and employ a qualified nurse to administer the service. The Federation of the Visually Impaired Persons in Finland has 16 regional secretaries distributed across the whole of the country, but their services cover the country unequally and do not reach every corner of Finland to the same degree, leaving northern and eastern regions, where distances between settlements are large, disadvantaged. Obviously, given the unequal coverage of the services in the country, improvements in caring for the blind and visually handicapped especially in remote parts of the country are still possible.

Although our conclusions of elevated suicide risks concerning the Finnish visually impaired (especially blind men of working age) may not be applicable to other countries and geographic localities, the finding that surgery to correct visual impairment due to cataract appears to improve the long-term survival of older persons [48] fits our observations. Special attention must be given to people during the period following the recognition of the visual impairment, because blind people appear to cope significantly better with being given a realistic prognosis of their future visual capacity than people with low vision and age-related visual deterioration. The latter tend to commit suicide earlier than the former, with the greatest difference between the two groups 4–14 years after the recognition of the visual impairment. In both groups, as with the reported increased suicide risk following a cancer diagnosis [49], the risk elevations decreased rapidly during the first year after diagnosis.

We recognize certain limitations of our study: firstly, comorbid disorders and earlier suicide attempts known to be generally correlated with depressive symptoms and hopelessness [8,33–35] were not part of our suicide data and could therefore not be considered in our analyses; secondly, social conditions with regard to marital status, education, income, and place of residence, although clearly of importance in the much larger data sets of, for example, sighted victims, remained unevaluated in our study, since permission to use them was not obtained and they, on account of the small data base covering blind and visually impaired victims, might not have yielded statistically relevant conclusions anyway. Complete data on the degree of visual impairment were available for all but one of the visually impaired subjects, an individual who we assigned to the low vision group given that the majority of the visually impaired belonged to that category. The overall aim of this first nationwide study focusing on the blind and visually impaired had been to examine whether their suicides differed with regard to gender, age, method and season from suicides committed by sighted people and to discuss possible differences and similarities between the two groups so that corrective measures might possibly be initiated.
